# Intravenous Immunoglobulin for Autoimmune Bullous Diseases: A Case Series from a Central European Referral Center

**DOI:** 10.3390/medicina59071265

**Published:** 2023-07-07

**Authors:** Maciej Marek Spałek, Monika Bowszyc-Dmochowska, Marian Dmochowski

**Affiliations:** 1 Autoimmune Blistering Dermatoses Section, Department of Dermatology, Poznan University of Medical Sciences, 60-355 Poznan, Poland; mdmochowski@ump.edu.pl; 2 Cutaneous Histopathology and Immunopathology Section, Department of Dermatology, Poznan University of Medical Sciences, 60-355 Poznan, Poland; m.bowdmo@wp.pl

**Keywords:** autoimmune bullous diseases, intravenous immunoglobulin, glucocorticosteroids

## Abstract

*Background and Objectives*: Autoimmune bullous diseases (AIBDs) may be treated with intravenous immunoglobulin (IVIG) infusions. This study aimed to evaluate the benefits and safety profiles of high-dose IVIG therapy in AIBD patients, as determined by clinical remission, the glucocorticosteroid-sparing effect, and adverse events at 12 months follow-up in a Central European university dermatology department setting. *Materials and Methods*: Our case series included 10 patients: five patients with pemphigus vulgaris, one with pemphigus herpetiformis, one with pemphigus foliaceus, one with bullous pemphigoid, two with epidermolysis bullosa acquisita. They underwent 4–12 monthly cycles of IVIG therapy at a dose of 2 g/kg per cycle. *Results*: The prednisone dosage reduction after 2, 6, and 12 months following the final IVIG course was 65.45%, 70.91%, and 76.37%, respectively. During the 12-month observation period, disease relapse was observed in 20% of patients, while others achieved complete or partial remission without or with minimal therapy. Side effects were seen in 80% of patients; they were transient and did not necessitate discontinuation of IVIG. *Conclusions*: IVIG demonstrates effectiveness as a treatment with a favorable safety profile. Nevertheless, its high cost remains a significant drawback, particularly in low-income countries. IVIG should be considered, especially in patients opposed to standard therapies or with contraindications to their use.

## 1. Introduction

Autoimmune bullous diseases (AIBDs), of which bullous pemphigoid (BP) is the most common in Europe, are a group of relatively rare, potentially life-threatening diseases caused by autoantibodies targeting adhesion molecules in the skin and/or mucous membranes, resulting in varying inflammatory responses [1]. AIBDs, including pemphigus vulgaris (PV), present numerous clinical peculiarities, which may delay therapies suitable for them [2].

Systemic glucocorticosteroids (GCS) remain the first-line, live-saving drugs in pemphigus; however, the high doses and prolonged courses required for disease control can lead to many dangerous side effects, including death [3]. Achieving disease remission with the lowest possible cumulative GCS dose is crucial, often necessitating the use of immunosuppressive agents such as azathioprine (AZA), mycophenolate mofetil (MMF), methotrexate (MTX), cyclophosphamide (CPH), cyclosporine (CSA), and dapsone (DP) [4,5]. Additional treatment options include rituximab (RTX), intravenous GCS (IGCS) pulses, immunoadsorption, plasmapheresis, and IVIG [6].

Human immunoglobulin has been used to treat people since 1952 when Bruton treated a child with recurrent pneumococcal infections, followed by Imbach et al. who used IVIG to treat a child with immune thrombocytopenia [7,8]. Due to its immunomodulating effect, it has found a place in the therapy of many autoimmune disorders in dermatology including PV, BP, pemphigus foliaceus (PF), mucous membrane pemphigoid, epidermolysis bullosa acquisita (EBA), dermatomyositis, systemic vasculitis, and systemic lupus erythematosus [9,10,11].

Adding IVIG to the standard therapy of PV (prednisone, immunosuppressive drugs) seems to lead to long-term remission without drugs, achieved through its ability to eliminate selected disease-specific IgG antibodies [12,13]. Moreover, IVIG can alter the expression of Fc receptors and influence the activation and regulation of T and B cells [8]. Furthermore, it has a very mild side effect profile [13]. The main disadvantage of IVIG therapy is its high cost and limited availability [14].

This study aimed to evaluate the effectiveness of the treatment of autoimmune bullous diseases with intravenous immunoglobulins during a 12-month observation period.

## 2. Materials and Methods

The medical records of patients diagnosed with AIBDs who underwent IVIG treatment at the dermatology department in Poznan between 2014 and 2022 were retrospectively analyzed. The study employed the following exclusion criteria:

- Patients who were receiving a prednisolone equivalent dosage of less than 10 mg/day prior to the first IVIG cycle, except for those with absolute contraindications to GCS,
- Patients who had fewer than 10 active lesions (such as blisters, erosions, or new areas of erythema) before the initial IVIG infusion,
- Patients for whom follow-up data was unavailable for at least one year after the completion of the last IVIG cycle,
- Patients with a history of hypersensitivity reactions to the tested drug,
- Patients diagnosed with IgA deficiency.

Following the application of these exclusion criteria, a total of 10 patients met the inclusion criteria and were included in the study.

The diagnoses of PV, PF, BP, EBA, and pemphigus herpetiformis (PH) were based on clinical signs, histological findings, and immunological studies: direct immunofluorescence (DIF), indirect immunofluorescence, and multiplex ELISA for IgG antibodies to desmoglein 1, desmoglein 3, BP 180, BP 230, envoplakin, and type VII collagen [15].

Patients included in the study were treated with IVIG infusions intravenously for at least four consecutive courses, with a maximum of twelve courses. The total dose of 2 g/kg per cycle was divided into five equal doses (400 mg/kg) and administered on five consecutive days in a hospital setting. Each cycle was repeated every 4 weeks. Blood pressure and urine production were monitored during each infusion. Before each cycle, a complete blood count, renal and liver function tests, and urine analysis were performed. Before the first cycle, serum immunoglobulin levels were also measured. The patients were followed up 2, 6, and 12 months after their last IVIG course.

We used the consensus described by Murrell DF et al. to define the control of disease activity [16]. A complete remission off therapy (CRNT) was defined as the absence of new and/or established lesions while the patient was not receiving systemic therapy for at least two months. If a patient did not have any lesions while receiving less than, or equal to, 10 mg/day prednisone or additional immunosuppressants, disease activity was classified as complete remission on minimal therapy (CRMT). Relapse ® of disease was defined as the appearance of 3 or more new lesions in a month that did not heal spontaneously within 1 week. A partial remission on minimal therapy (PRMT) was defined as the presence of transient new lesions that healed within one week while the patient was receiving minimal therapy.

## 3. Results

In total, 10 patients (5 with PV, 1 with PH, 1 with PF, 1 with BP, and 2 with EBA) were included in our case series study. There were five females and five males aged 58.5 ± 14.7 (mean ± SD) years, with an age range of 30 to 78 years. The time from disease diagnosis to the start of IVIG therapy was a minimum of 0.3 years and a maximum of 6.5 years, with a mean of 1.76 years ± 1.85.

General patient characteristics are shown in Table 1.

The mean initial prednisone equivalent dosage was 30.56 mg ± 21.14, and it was gradually tapered in patients with controlled disease. Following the first two IVIG cycles, the prednisone equivalent dosage was reduced by approximately half, and subsequent tapering continued until the cessation of IVIG treatment. However, some patients required an increase in dosage to effectively manage the disease. Two months after completing all IVIG cycles, the mean prednisone equivalent dosage was 10.56 mg ± 4.9. At 6 months, it further decreased to 8.89 mg ± 6.5, and at 12 months, it reached 7.22 mg ± 7.49. Therefore, the reductions in dosage were 65.45%, 70.91%, and 76.37% at 2, 6, and 12 months, respectively.

**Figure 1:**
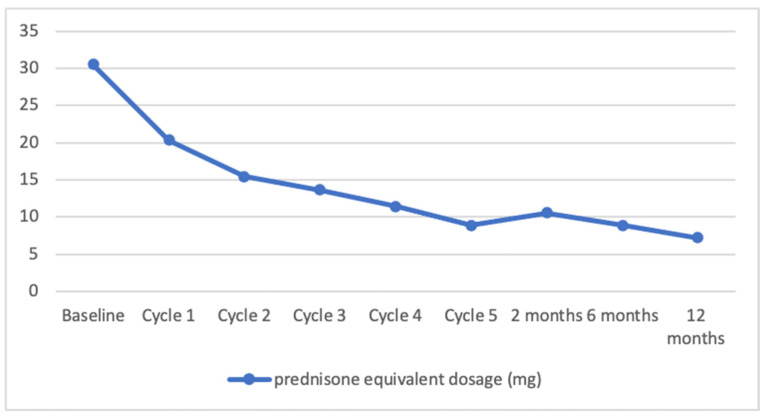
Decrease in mean prednisone equivalent dosage during intravenous immunoglobulin (IVIG) therapy in autoimmune bullous diseases.

Figure 1 presents the data regarding patients’ response to IVIG therapy during the first five cycles and the 1-year follow-up period.

Prior to the administration of IVIG therapy, patients were treated with oral GCS (OGCS), various immunosuppressants, RTX, and DP. Some patients required additional therapy after IVIG infusions. Patient #4, a 38-year-old with PV, received DP 50 mg/day, while patient #7, a 56-year-old female with PH, was taking AZA 50 mg/day. Patient #9, a 60-year-old female with EBA, had two infusions of RTX 1 g each and received methylprednisolone pulse intravenously (1 g daily over 3 days).

**Figure 2:**
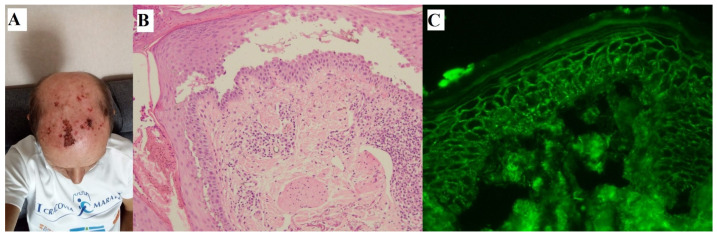
A noteworthy 69-year-old patient with cutaneous PV (#1), in whom the value of IgG antibodies to desmoglein 3 with multiplex ELISA at the time of diagnosis was highly elevated 5.50 (cut-off value = 1). Erosions covered with necrotic crusts on the frontal-parietal surface of the head (**A**). Suprabasilar separation with acantholytic keratinocytes seen in H + E histology (original objective magnification ×20) (**B**). IgG4 (++) pemphigus deposits with DIF visualized using a short-arc mercury-lamp-operated microscope (original objective magnification ×40) (**C**). Despite a complete clinical remission after IVIG therapy, the value of IgG antibodies to desmoglein 3 was still elevated (value 3.75) at follow-up 17 months after the diagnosis.

In our calculations of prednisone equivalent dosage, we excluded one patient with PV (#1) (Figure 2) who, due to contraindications (active hepatitis C viremia treated with glecaprevir/pibrentasvir), could not start GCS. Patient #10, a patient with BP, received IGCS before IVIG infusions; however, he developed Mallory–Weiss syndrome, which contraindicated the continuation of GCS, and IVIG therapy was initiated.

In the case of patient #7 with PH, IVIG proved to be a life-saving procedure. The patient experienced exacerbation of her disease, triggered by an incarcerated Meckel’s diverticulum, which led to bowel obstruction and feculent vomiting. Patient #9 with EBA experienced a direct exacerbation of the disease involving mucosal tissues immediately after IVIG therapy, resulting in the need for a tracheostomy. Despite receiving RTX infusion, there was no improvement observed at the 3-month follow-up. As a result, the patient received methylprednisolone pulse (1 g daily over 3 days), which eventually brought the disease under control.

We also encountered a patient with severe PV specifically involving the conchae of the auricles. This patient underwent five IVIG cycles, which resulted in complete remission of PV symptoms. However, due to the unavailability of a comprehensive 1-year follow-up period, it was not feasible to analyze further this patient according to our study design.

**Figure 3:**
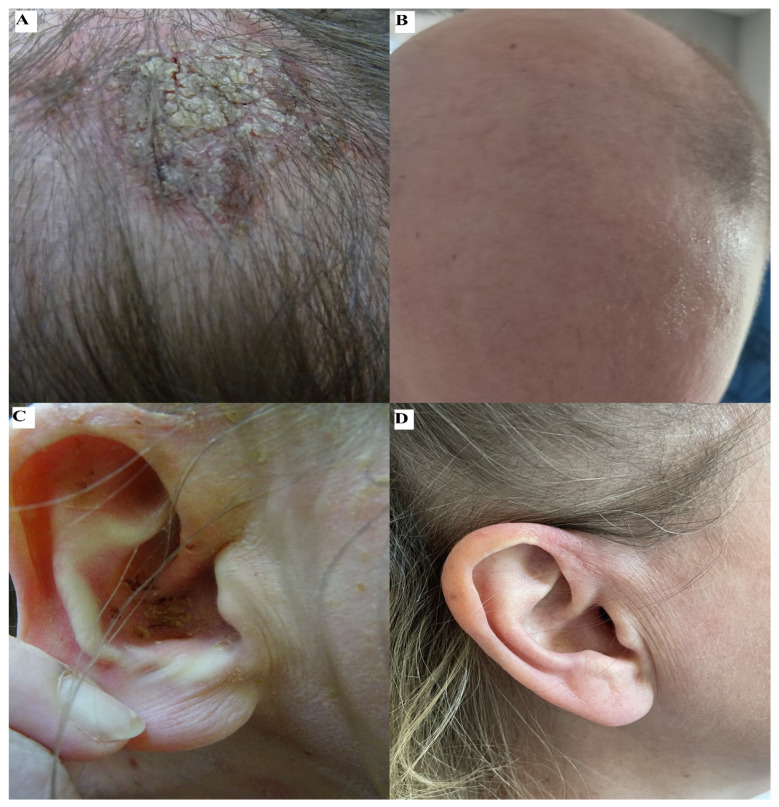
Two representative patients with PV. A middle-aged male, followed-up for more than one year after IVIG treatment, who initially exhibited a greenish crusted lesion on an erythematous erosive base localized on the parietal region of the scalp (**A**). The lesion healed following a combined therapy, which included 12 IVIG courses (**B**). A middle-aged female, followed-up for less than one year after IVIG treatment, initially presented with a yellowish crusted erosion localized in the concha of the right auricle (**C**). This lesion healed following a combined therapy, which included 5 IVIG cycles (**D**).

The treatment effects of the aforementioned patient and patient #4 are depicted in Figure 3.

Initially, following the first treatment cycle, patients exhibited notable clinical improvement. However, over the subsequent 2 months of treatment, a decline in treatment response became evident. Specifically, relapse of the disease was observed in 50% of patients at the 2-month mark, which subsequently decreased to 20% after a 12-month period. Additionally, at the 2-month evaluation, 10% of patients achieved complete remission with no treatment (CRNT), while the number increased to 20% during the 12-month follow-up period. Partial remission with no treatment (PRNT) and complete remission with minimal treatment (CRMT) were observed in 40% and none of the patients, respectively, at the 2-month observation, but these percentages changed to 50% and 10%, respectively over time. 

**Figure 4:**
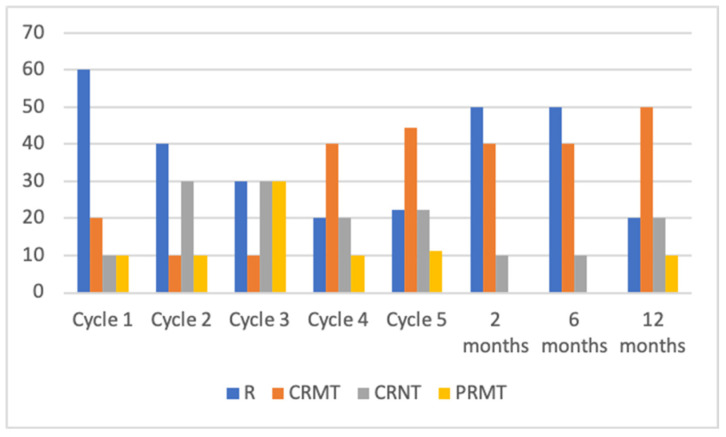
Percentage distribution of treatment effects during intravenous immunoglobulin (IVIG) therapy in AIBDs; CRNT, complete remission off (no) therapy; CRMT, complete remission on minimal therapy; PRMT, partial remission on minimal therapy; R, relapse.

Data about patients’ response to IVIG therapy (first five cycles and 1-year follow-up) is shown in Figure 4.

Side effects were observed in 80% of the patients, demonstrating a transient nature that did not warrant the discontinuation of IVIG therapy. Among these, the most prevalent side effect was leukopenia, which manifested after the first infusion in patients #1 and #5, after the third cycle in patient #3, and following the seventh infusion in patient #10. During the initial IVIG infusion, three patients (#2, #4, #5) experienced hypotension, prompting a reduction in the infusion rate. Similarly, in the case of two patients (#2 and #8) who experienced headaches after their first IVIG cycle, the infusion rate was accordingly decreased. Over the 1-year follow-up period, two patients (#3 and #8) developed fungal infections in the oral cavity, which were effectively managed using systemic antimycotics. Furthermore, patient #10 reported experiencing nausea shortly after the second infusion, which spontaneously resolved within a few hours. 

Patients’ treatment characteristics are shown in Table 2.

## 4. Discussion

This study was subject to certain limitations, including a small sample size. The rarity of AIBDs and the high cost associated with IVIG therapy contribute to the limited availability of participants. Additionally, the heterogeneity of our patient group, consisting of individuals diagnosed with various AIBDs, poses a challenge. However, it should be noted that IVIG treatment promotes the accelerated degradation of pathological autoantibodies produced during the course of various AIBDs, thereby exhibiting a similar mechanism of action regardless of the specific diagnosis [9]. Furthermore, retrospective analysis of photographic archives and reliance on medical records for data description represent potential weaknesses of our study. Therefore, further investigations involving larger cohorts of patients with AIBDs are warranted to comprehensively evaluate the effectiveness and safety of IVIG therapy. 

In 2009, Amagai et al. conducted the first randomized double-blind trial, demonstrating the efficacy of high-dose IVIG in the therapy of pemphigus. In 2017, the same authors designed a similar study in patients with BP, reaching the same conclusion [17,18]. In our study, we have presented clinical responses and 1-year follow-ups after the last IVIG infusion. Our research included patients with various autoimmune bullous disorders resistant to immunosuppressants and GCS, who were unable to achieve long-term remission. 

Of our participants, 80% were able to maintain partial and complete remission at 12 months, of whom 60% required minimal therapy. This long-term remission is likely associated with IVIG’s ability to modulate the function of Fc receptors and affect the production of pathological autoantibodies [9,19]. Moreover, there was a noticeable reduction in GCS dosage, reaching 65.45% in the second month after the last IVIG course and 76.37% at 12 months. These findings regarding the GCS-sparing effects of IVIG are supported by the existing literature and are consistent with our study [20,21,22]. However, during the 2-month follow-up, five patients experienced disease relapse, and ultimately, two participants with PV and PF, aged 71 and 78 years, respectively, did not achieve clinical remission within the 12-month timeframe. Kridin et al. found that an age ≥65 years at diagnosis was a significant factor associated with fatal outcomes in patients with pemphigus, which could be one of the reasons why our patients did not respond to treatment [23].

Furthermore, in 2016 Enk et al. promoted IVIG as second-line therapy in AIBDs refractory to standard therapy. They also recommended considering IVIG in patients previously treated with RTX in whom disease control was not attained [24]. One patient from our study with PV was previously treated with RTX and did not achieve clinical remission. He underwent five IVIG courses and after 12 months, received CRMT which is consistent with the conclusions of Enk et al. Moreover, IVIG may be used as a monotherapy with a good long-term effect, as demonstrated by our patient who had contraindications for GCS therapy and achieved complete remission without any treatment [25,26,27].

Due to the rarity of EBA, there is no specific treatment dedicated to this disease. IVIG was proposed by Miyamoto et al. as part of its therapy [28]. Sesarman et al. showed, in an experimental model, that IVIG can decrease the number of pathogenic autoantibodies by blocking the neonatal Fc receptor in EBA [29]. Justin M. et al. described a case of a man diagnosed with EBA who was treated with DP and IVIG infusions, achieving complete remission after a 1-month follow-up, while Campos et al. described a case of women with EBA successfully treated with IVIG infusions with full remission, although without follow-up [30,31]. In 2012, Ahmed and Gurcan conducted research involving 10 EBA patients unresponsive to conventional therapy, who received an average of 23.1 IVIG cycles over a mean period of 38.8 months. All patients achieved complete clinical remission without any additional treatment and were observed for a long period, ranging from 29 to 123 months [32]. A meta-analysis conducted by Iwata et al. included over 1000 EBA patients and demonstrated that IVIG therapy was statistically significantly and associated with complete remission [33]. The abovementioned studies correlate with our two patients with EBA, who had CRNT and CRMT 12 months after the final IVIG course, following treatment with 5 IVIG infusions and 12 infusions, respectively. Data from other studies suggest that increasing the number of IVIG courses may be beneficial for the second patient in terms of achieving complete remission instead of partial remission.

Adverse reactions accompanying IVIG infusions occur, on average, in 20% of patients, and the majority of them are mild [34]. Flu-like symptoms and headaches appear to be the most common, ranging from mild to moderate severity. However, it should be noted that severe side effects can occur, including thrombotic events, aseptic meningitis, and hemolysis [35]. In our study, the reported adverse events during the 12-month follow-up period were transient and mild; thus, discontinuation of IVIG therapy was not necessary. Patients with fungal infections received treatment with systemic or topical antimycotics, while slowing down the infusion rate proved helpful in managing headaches, hypotension, and nausea.

Leukopenia emerged as the most frequently observed side effect in our patient cohort. According to Cicha et al., out of 35 patients who received either low or high doses of IVIG, a decrease of ≥10% in absolute leukocyte counts was observed in 30 individuals. Among the factors implicated in the occurrence of leukopenia following IVIG administration, two mechanisms can be discerned: the production of antibodies targeting neutrophils and the formation of immunoglobulin aggregates, which modulate the function of surface receptors on neutrophils, triggering oxidative burst and degranulation, resulting in a decrease in the number of circulating cells [36]. In our cohort of patients who experienced leukopenia, we conducted daily monitoring of the full blood count and recorded that leukopenia resolved spontaneously in all cases. Therefore, from our observations, IVIG has a good safety profile and adverse reactions are manageable.

## 5. Conclusions

In conclusion, IVIG therapy was able to maintain a 1-year remission in patients with various AIBDs refractory to first-line treatment, however, it was not efficient in all cases. The safety profile of IVIG is good, as the therapy induced only transient adverse effects which were not the reason for the cessation of therapy. It seems to be beneficial to administer more IVIG courses to older patients, as their response to therapy may be weaker. High cost and limited availability, particularly in low-income countries, are the main disadvantages of IVIG therapy. Nevertheless, our case series study suggests that IVIG can be taken into consideration for the treatment of AIBDs, especially considering its favorable safety profile, even in low-income countries.

## Figures and Tables

**Table 1:** Patient characteristics.

Patient	Gender/Age (Years)	Disease Type	No. of IVIG Cycles	Duration of Disease Prior to IVIG Therapy (Years)
1	M/69	PV	5	0.5
2	M/30	PV	4	0.5
3	M/54	PV	5	3.0
4	M/38	PV	12	0.5
5	F/78	PV	6	1.5
6	F/71	PF	5	3.0
7	F/56	PH	12	6.5
8	F/55	EBA	6	0.5
9	F/60	EBA	12	1.0
10	M/74	BP	9	0.75

PV, pemphigus vulgaris; PH, pemphigus herpetiformis; PF, pemphigus foliaceus; EBA, epidermolysis bullosa acquisita; BP, bullous pemphigoid.

**Table 2:** Patients’ treatment characteristics.

Patient	Previous Therapies	Prednisone Equivalent Dosage before IVIG (mg)	Prednisone Equivalent Dosage (mg) after IVIG Post			Additional Treatment	Side Effects	Control of Disease Activity after		
			2 Months	6 Months	12 Months			2 Months	6 Months	12 Months
1	None	0	0	0	0	None	Leukopenia	CRNT	CRNT	CRNT
2	OGCS	80	10	5	5	None	Headaches, hypotension	CRMT	CRMT	CRMT
3	OGCS, RTX, DP	10	10	10	5	None	Fungal infection	R	R	CRMT
4	OGCS, AZA, DP, CSA, IGCS	20	15	15	5	DP 50 mg/day	Hypotension	R	R	PRMT
5	OGCS, MMF, CPH, DP, AZA	15	20	20	20	None	Leukopenia, hypotension	R	R	R
6	OGCS, DP, CPH	20	10	20	20	None	Leukopenia	CRMT	R	R
7	OGCS, AZA, DP	40	10	5	0	AZA 50 mg/day	None	CRMT	CRMT	CRMT
8	OGCS	20	10	5	0	None	Headache, fungal infection	CRMT	CRMT	CRNT
9	OGCS, DP, IGCS, AZA, MTX, CPH, PPH	20	10	10	10	RTX 1 g two times, IS 3 pulses 1 g each	None	R	R	CRMT
10	DP, IGCS	50	40	0	0	DP 50 mg/day	Nausea, leukopenia	R	CRMT	CRMT

CRNT, complete remission off (no) therapy; CRMT, complete remission on minimal therapy; PRMT, partial remission on minimal therapy; R, relapse; RTX, rituximab; OGCS, oral glucocorticosteroids; DP, dapsone; AZA, azathioprine; MTX, methotrexate; CPH, cyclophosphamide; IGCS, intravenous glucocorticosteroids; CSA, cyclosporine; MMF, mycophenolate mofetil; PPH, plasmapheresis.

## Data Availability

The datasets generated and/or analyzed in the current study are not publicly available due to data privacy, but are available from the corresponding author on reasonable request.
